# Efficacy and Effectiveness of Extracorporeal Shockwave Therapy in Patients with Myofascial Pain or Fibromyalgia: A Scoping Review

**DOI:** 10.3390/medicina58081014

**Published:** 2022-07-28

**Authors:** Marco Paoletta, Antimo Moretti, Sara Liguori, Giuseppe Toro, Francesca Gimigliano, Giovanni Iolascon

**Affiliations:** 1 Department of Medical and Surgical Specialties and Dentistry, University of Campania “Luigi Vanvitelli”, 80138 Naples, Italy; marco.paoletta@unicampania.it (M.P.); sara.liguori@unicampania.it (S.L.); giuseppe.toro@unicampania.it (G.T.); giovanni.iolascon@unicampania.it (G.I.); 2 Department of Physical and Mental Health and Preventive Medicine, University of Campania “Luigi Vanvitelli”, 80138 Naples, Italy; francesca.gimigliano@unicampania.it

**Keywords:** extracorporeal shockwave therapy, fibromyalgia, myofascial pain syndromes, musculoskeletal pain, rehabilitation, trigger points, neck pain, pressure pain threshold

## Abstract

Myofascial pain syndrome (MPS) and fibromyalgia (FM) are underestimated painful musculoskeletal conditions that could impact function and quality of life. A consensus about the most appropriate therapeutic approach is still not reached. Considering the long course of the diseases, prolonged assumption of drugs, such as NSAIDs and pain killers, could increase the risk of adverse events, often leading affected patients and physicians to prefer non-pharmacological approaches. Among these, radial and focused extracorporeal shock waves therapies (ESWT) are widely used in the management of painful musculoskeletal conditions, despite the fact that the mechanisms of action in the context of pain modulation should be further clarified. We performed a scoping review on PubMed using Mesh terms for analyzing the current evidence about the efficacy and effectiveness of ESWT for patients with MPS or FM. We included 19 clinical studies (randomized controlled trials and observational studies); 12 used radial ESWT, and 7 used focused ESWT for MPS. Qualitative analysis suggests a beneficial role of ESWT for improving clinical and functional outcomes in people with MPS, whereas no evidence was found for FM. Considering this research gap, we finally suggested a therapeutic protocol for this latter condition according to the most recent diagnostic criteria.

## 1. Introduction

Myofascial pain syndrome (MPS) and fibromyalgia (FM) are musculoskeletal (MSK) conditions that significantly affect function and quality of life (QoL). Myofascial pain syndrome has been defined as a regional pain characterized by the presence of one or more myofascial trigger points (MTrPs), or ‘taut bands’, that are a limited number of hyperirritable muscle fibers organized in nodules that can cause spontaneous and referred pain on palpation [1]. The pathophysiology of myofascial pain is not well defined [2]. It has been hypothesized that the sensitization of low-threshold mechanosensitive afferents triggered by a local dysfunction of the motor endplates in the MTrPs area [3] is one of the main pathogenetic mechanisms, as also suggested by the local increase in inflammatory mediators, neuropeptides, cytokines and catecholamines in the tissue around the active MTrPs [4]. These metabolites may contribute to nerve dysfunction, particularly autonomic and sensory small fiber, leading to local vasoconstriction and decreased blood flow as well as referred pain, allodynia, and hyperalgesia [2]. This condition seems to hesitate in characteristic findings at ultrasound evaluation, where trigger points appearing as hyperechoic (hypoperfused) spots in hypoechoic areas [5].

Fibromyalgia is a chronic disease characterized by widespread MSK pain with specific limited soft tissue areas of hyperalgesia and/or allodynia (i.e., tender points). Moreover, affected patients complain of fatigue, sleep disorders, and other somatic and cognitive symptoms [6]. It should be underlined that MPS and FM are often characterized by challenging differential diagnoses because of possible overlaps in pain distribution, duration of symptoms, and physical findings.

Several interventions have been proposed to treat FM and MPS, such as drug therapy, exercise, physical therapy, acupuncture, and needling (dry needling, trigger point injection). However, the most appropriate and effective approach for these conditions is still debated [7]. Extracorporeal shockwave therapy (ESWT) is a non-invasive physical modality used for several painful MSK disorders [8]. This intervention should exert numerous biological effects with potential clinical benefits in patients with MSK diseases. It was hypothesized that the main biological effect on treated tissue by ESWT is an increase in the permeability of cell membranes and the release of several molecules stimulating tissue regeneration [9], such as Vascular Endothelial Growth Factor (VEGF), Fibroblast Growth Factor (FGF) and the activation of the endothelial nitric oxide synthase (eNOS) with angiogenic effects [10]. Finally, ESWT has an important role in pain relief by modulating the release of anti-inflammatory mediators and endorphins that activate descending inhibitory system [11].

The aim of this scoping review is to summarize current evidence about the efficacy and effectiveness of ESWT in patients with FM or MPS.

## 2. Materials and Methods

In performing this scoping review, we followed the PRISMA-ScR (Preferred Reporting Items for Systematic Reviews and Meta-Analyses Extension for Scoping Reviews) guidelines [12].

### 2.1. Search Strategy

We planned a search on PubMed (Public MedLine, run by the National Center of Biotechnology Information, NCBI, of the National Library of Medicine of Bethesda, Bethesda, MD, USA) with ad hoc search strings with selected keywords for FM, MPS, and ESWT (Table 1).

### 2.2. Study Selection

According to the study objective, we defined the characteristics of the sources of evidence, considering for eligibility any research published in the medical literature until 31 December 2021 and including only those in the English language and conducted on humans (Table 2).

### 2.3. Data Extraction and Quality Assessment

Clinical research, including interventional (randomized or non-randomized controlled clinical trials) and observational studies, were selected. Research findings from each included study were qualitatively analyzed.

## 3. Results

**Figure 1:**
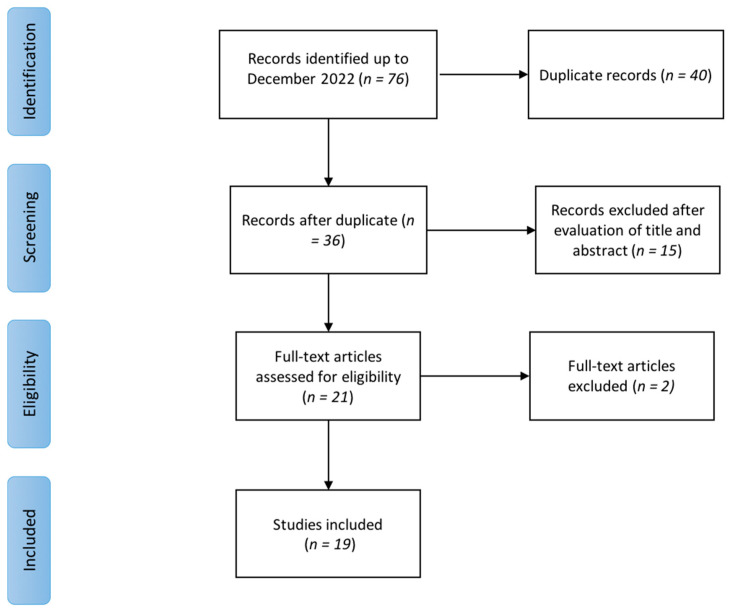
Flow diagram of the literature review process.

Seventy-six items were initially found. After duplicate removal, 35 records remained. We screened them on the basis of titles and abstracts for inclusion/exclusion criteria, and 14 studies were excluded. After full-text reading, we excluded another two articles because the authors did not specify the type of ESWT used. Finally, we included in this review 19 studies published between 2012 and 2021. None of the trials involving people with FM met the eligibility criteria. Among those including people with MPS, 12 used radial ESWT (rESWT), and 7 used focused ESWT (fESWT). Figure 1 summarizes the selection process of the included papers. Table 3 and Table 4 report the characteristics and main findings of the included studies.

### 3.1. Radial ESWT

Of the 12 studies investigating the effectiveness of rESWT for the management of patients with MPS, 10 are RCTs (1 pilot) [13,14,15,16,17,18,19,20,21,22], one is a case-control study [23], and one is a retrospective study [24] (see Table 3 for further details). Two RCTs compared rESWT with laser therapy reporting a reduction in pain and disability with both modalities [13,14]. Two RCTs compared rESWT with ultrasound therapy (US), showing that rESWT was equally effective to US in reducing pain, reducing disability, and improving QoL and that both techniques were more effective than sham treatment or exercise alone [15,16]. Another RCT compared rESWT to a combination of hot packs, Trans Cutaneous Electrical Nerve Stimulation (TENS), and US and showed that rESWT was more effective in reducing pain and improving sleep quality, disability, depression, and QoL [17]. Taheri et al. 2021 compared rESWT with phonophoresis and reported that both techniques effectively decreased pain and neck disability with the superiority of rESWT [18]. Three RCTs compared rESWT with dry needling (DN) reporting that both interventions were effective in reducing pain and disability [19,20,21]. One of these trials reported that DN could be associated with post-treatment soreness [19]. Another RCT compared rESWT with corticosteroid trigger point injection (TPI) and reported that after one month of treatment, rESWT was more effective in reducing pain and disability and improving QoL [22].

### 3.2. Focused ESWT

Of the seven studies investigating the efficacy and effectiveness of fESWT, five were RCT [25,26,27,28,29], and two were retrospective studies [30,31] (see Table 4 for further details). Most RCTs [25,26,27,28] investigated the efficacy of fESWT on neck pain, particularly in the upper trapezius, while only one study [29] analyzed the treatment on gastrocnemius–soleus muscle. Ji et al. [26] and Park et al. [27] compared fESWT with ineffective and low energy fESWT, respectively, reporting a significant improvement in pain and NDI score in the intervention groups, although using different treatment protocols. At the same time, Kamel et al. [28] found that combined treatment with 1% topical diclofenac gel and fESWT significantly improved pain, neck ROM, and PPT compared to 1% topical diclofenac gel only in the same population. The results of the study by Jeon et al. [25] suggest no significant between-group difference in terms of pain measures and neck mobility 1 week after the first and the third treatment. Finally, Moghtaderi et al. [29] reported that the treatment of gastroc–soleus trigger points in patients with plantar fasciitis showed better results on pain (VAS) and activity (modified Roles and Maudsley score) at 8 weeks after the last treatment compared to control group where only heel region was treated.

Two observational studies investigated the effectiveness of fESWT in the treatment of MPS in the low back (quadratus lumborum) [30] and the upper part of the unilateral trapezius [31]. Hong et al. [30] compared an interventional protocol of fESWT with corticosteroids (CS) TPI. Authors found that fESWT was more effective than control in reducing pain and increasing PPT at the end of treatment and at 1-month follow-up, but no difference was found for disability measures (ODI, RM score, QBS). Yalcin [31] compared ESWT plus exercise with kinesiotaping (KT) plus exercise and exercise, only reporting better results for patients receiving ESWT plus exercise in terms of pain and contralateral neck lateral flexion.

## 4. Discussion

To our knowledge, this is the first PRISMA-driven scoping review aiming to investigate the efficacy and effectiveness of ESWT in the treatment of patients with MPS or FM.

First of all, we must stress that although there are numerous studies dealing with the efficacy of ESWT in patients with MPS in the literature, no paper has aimed to evaluate this intervention in people with FM.

Of note, starting from an overview of included studies, a substantial issue concerned the heterogeneity of treatment protocols, particularly in terms of the number of sessions, intervals between sessions, number of SW administered per TrP, and intensity. About rESWT, shock waves number ranged from 1000 to 4500 (most authors administered 2000 SW; Aktürk et al., 2018, Luan et al., 2019, Rahbar M. et al., 2021, Taheri P. et al., 2021). This intervention was usually provided once a week for 3 weeks or sometimes for four sessions, while only Gezginaslan et al. carried out seven sessions with 3-day intervals. For fESWT, the number of SW used ranged from 1000 to 3000, while the intensity ranged from 0.056 to 0.25 mJ/mm^2^. Considering the number of sessions, authors generally performed one session per week for 3 weeks (Jeon et al., 2012, Moghtaderi et al., 2014, Hong et al., 2017, Kiraly et al., 2018, Ümit Yalçın 2021).

Among studies included in our review (15 RCTs, four observational studies), 16 compared ESWT to other interventions, while two studies compared this intervention to sham ESWT. Moreover, in an observational retrospective study, no treatment was administered to the control group.

Observational studies comparing ESWT to another intervention reported that both focused and radial modalities were more effective than TPI, KT, and physical agents in MPS patients in terms of pain, mobility, and disability.

On the other hand, results from clinical trials investigating the efficacy of ESWT in MPS people are conflicting. In the RCTs comparing ESWT versus placebo (sham, ineffective ESWT for intensity or application site), both radial and focused modalities seem to significantly improve pain in people with MPS.

Regarding evidence about rESWT in comparison with DN, laser therapy combined with stretching, and therapeutic exercise, no significant differences were reported in terms of pain relief and disability, while rESWT seemed significantly more effective than TPI or a combination of physical agents (HP + TENS + US therapy or US therapy + hot pack) in terms of improvements of pain, fatigue, depression, sleep quality, disability, and QoL in patients with MPS.

RCTs investigating the efficacy of fESWT versus other interventions reported that this treatment modality was not better than TPI combined with TENS in terms of pain relief and mobility, while it was more effective than laser therapy in improving pain, disability, and QoL in patients with trapezius MPS. Moreover, the same intervention significantly improved pain and mobility compared to topical NSAIDs.

It was hypothesized that several mechanisms involved in the pathogenesis of clinical manifestations of MPS and FM might be addressed by different ESWT modalities.

Myofascial pain syndrome was described as muscle pain in different body regions reproduced by pressure on TrP, which are localized hardenings in skeletal muscle tissue. This condition may originate from muscular injury due to intense contractions or repetitive low-intensity overload, inducing an excessive release of acetylcholine by the neuromuscular endplate [32]. This event triggers a prolonged depolarization of muscle fibers, increasing calcium release from the sarcoplasmic reticulum and maintaining contraction with the formation of a so-called “knot”, which compresses local capillaries producing ischemia [33]. Ischemia, in turn, furtherly damages the dysfunctional endplate as well as induces the release of inflammatory mediators such as bradykinin, prostaglandins, serotonin, and histamine, leading to peripheral sensitization with hyperalgesia and allodynia [34].

On the contrary, pathogenic mechanisms underlying FM are still unclear. The characteristic tender points are considered as areas of tenderness symmetrically located in specific body parts that do not cause referred pain after stimulation. Recent studies suggest that various agents acting on central (psychological and cognitive-emotional factors) and peripheral nervous systems, including small nerve fibers (inflammatory mediators), can lead to neuromorphological modifications and pain dysperception [35]. Small fiber neuropathy can impair small blood vessels’ function through upregulation of α-adrenergic receptors and altered neuropeptide responses. This mechanism could explain impaired skeletal muscle perfusion, pain, and fatigue in patients with FM [36].

Considering that FM and MPS share some clinical and pathophysiological features, there might be a rationale for using physical agents, including ESWT, in the management of these conditions. Indeed, ESWT has documented effects on several MSK disorders, including the stimulation of angiogenesis with consequently improved perfusion of ischemic tissues.

**Figure 2:**
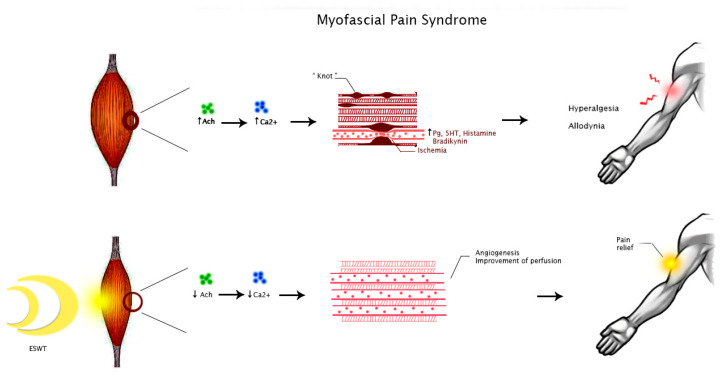
Hypothesized mechanisms of action of ESWT in people with MPS. Abbreviations: Ach, acetylcholine; Ca^2+^, calcium ion; Pg, prostaglandins; 5HT, 5-hydroxytryptamine.

**Figure 3:**
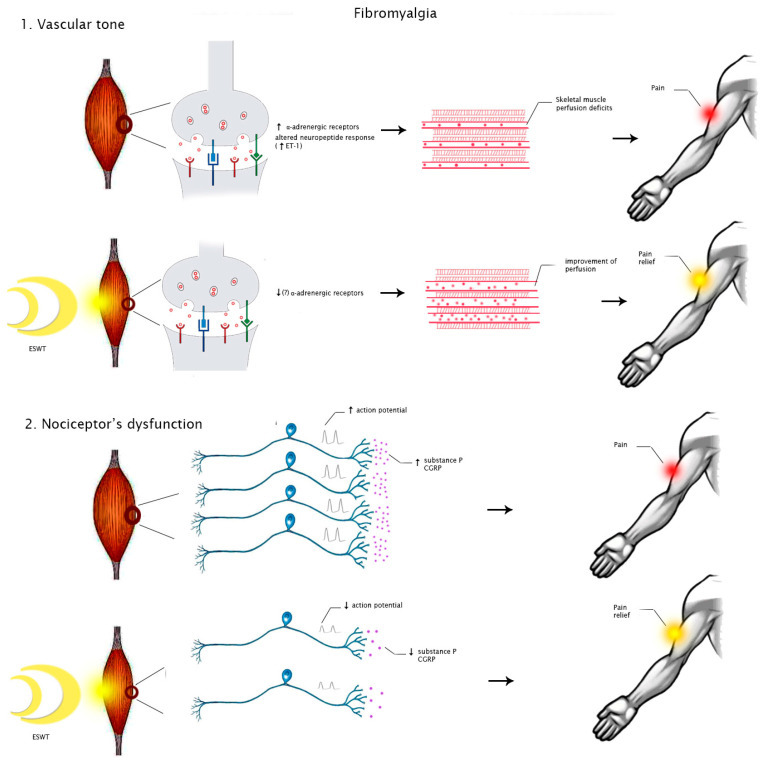
Hypothesized mechanisms of action of ESWT in people with FM. Abbreviations: ET-1, endothelin 1; CGRP, calcitonin gene-related peptide.

However, the biological effects of SW targeting pathogenic mechanisms of FM and MPS are still unclear. It is possible to speculate that ESWT may modulate ion influx, particularly of calcium, with consequent improvement of perfusion and promoting angiogenesis. These events might reduce local ischemia, enhancing tissue healing [37]. Moreover, this intervention seems to directly modulate nociception by producing a transient dysfunction of the nociceptor action potential [38]. Therefore, these mechanisms might justify the clinical benefits of this treatment on pain relief in people with MPS or FM (Figure 2 and Figure 3).

Despite our scoping review being the first comprehensive analysis of the role of both ESWT modalities in MPS and FM, some years ago, Ramon et al. already published an article dealing with this topic, proposing an ESWT protocol for a small cohort of FM patients [33]. In particular, the authors suggested performing from 1000 to 1500 SW for each of the three most painful points selected. Therefore, the patient should receive from 3000 to 4500 SW overall. However, this paper dates back before the publication of the new diagnostic criteria for FM, where pain must be present in at least four or five body regions [39]. If we applied this protocol to FM patients according to new diagnostic criteria, this approach would be too intense, thus compromising treatment compliance. We propose, according to available treatment protocols for MPS, that the suggested number of SW (3000 for fESWT, 4500 for rESWT) should be equally distributed for each painful region (i.e., 600–900 SW for five regions or 750–1100 SW for four regions, respectively) with SW intensity tailored according to patient tolerability. However, the evidence gaps about the minimum number of SW and ESWT intensity to obtain clinical benefits in different MSK disorders still persist. Therefore, further studies are needed to clarify the role and the best ESWT modality and parameters for the treatment of patients affected by MPS and FM.

## 5. Conclusions

Myofascial pain syndrome and FM are two complex conditions requiring challenging management. By considering the hypothesized pathophysiological mechanisms, the administration of ESWT was proposed for improving pain and disability in patients affected by these conditions, particularly MPS. Indeed, our scoping review suggests that ESWT could have a role in relieving pain and improving functional outcomes by modulating biological mechanisms of pain, inflammation, and angiogenesis in MPS. However, our results show that a widely accepted therapeutic schedule for both radial and fESWT has not been defined so far. Finally, considering the lack of evidence about the use of ESWT in people with FM, we proposed a new treatment protocol, based on the most recent diagnostic criteria taking into account patients’ tolerability, that needs to be investigated in future trials.

## Figures and Tables

**Table 1:** Search strategy.

(“Extracorporeal Shockwave Therapy”[Mesh] or “High-Energy Shock Waves”[Mesh]) and (“Fibromyalgia”[Mesh] or “Myofascial Pain Syndromes”[Mesh])
**“shockwave” and “myofascial”**
**“shock wave” and “myofascial”**
**“shockwave” and “myofascial pain”**
**“shock wave” and “myofascial pain”**
**“shock wave” and “fibromyalgia”**
**“shockwave” and “fibromyalgia”**

**Table 2:** Eligibility criteria.

**Inclusion criteria** Clinical studies (interventional and observational) about the efficacy and effectiveness of radial or focused ESWT on: o Fibromyalgia or o Myofascial Pain Syndrome
**Exclusion criteria** − Meta-Analysis − Systematic Reviews − Review Articles − Conference abstracts and editorials

**Table 3:** Main characteristics and key findings of the included studies about Radial ESWT for myofascial pain syndrome.

Author, Year	Study Design	Site of Application	Sample Size: Total (Group)	Intervention (ESWT Protocol)	Control	Outcomes	Timing	Main Findings
Taheri et al. 2016 [13]	RCT	Neck, shoulder (upper trapezius)	46 (26 intervention group, 20 control group)	SW: 1000 pulses EFD: 3 J/m^2^ and 10 Hz + stretching + not-specified drugs T: once a week for 3 weeks	Laser therapy (Indolaser device, type Ga-AL-As with 6 J/cm^2^, average power 100 mW, for total of 3 min on each spot for 10 sessions) + stretching + not-specified drugs	pain (VAS 1–10); disability (NDI; SPADI)	T0: baseline T1: at 5 weeks T2: at 7 weeks	Both rESWT and laser therapy proved to be effective in reducing pain and improving disability. Laser therapy showed statistically significant higher benefits at VAS and NDI compared to rESWT only at 5 weeks follow-up.
Kiraly et al. 2018 [14]	RCT	Neck, shoulder (upper trapezius)	61 (30 intervention group vs. 31 control group)	SW: 1000 + 1000 EFD: 1.5 bar and 10 Hz, 0.25 mJ/mm^2^ aroune the TP, subsequently 2 bar 10 Hz 0.25 mJ/mm^2^ on TP T: once a week for 3 weeks	Laser therapy (soft laser treatment daily for 15 days with 2000 Hz, 800 mW, 3 J/cm^2^ for 2 min on palpable trigger points and with 5000 Hz, 2000 mW, 9 J/cm^2^, for 2 min on trapezius muscles and trigger points	pain (VAS 0–100); disability (NDI); QoL (SF-36)	T0: baseline T1: at 3 weeks T2: at 15 weeks	Both rESWT and laser have proven to effectively improve pain tolerance, neck functionality, and quality of life, but the clinical effectiveness of rESWT was found to be higher.
Akturk et al. 2018 [15]	RCT	Neck	60 (20 rESWT, 20 US, and 20 sham rESWT)	SW: 2000–3000 shock/session, 200–400 shocks/trigger point EFD: 1.6−3.0 bar, 200–400 shocks/trigger point T: maximum 3 min/session, with at most 3-day intervals between sessions for a total of 4 sessions	US treatment for 2 weeks for 5 days a week (10 sessions), each session lasting for 5 min at a dose of 1.5 w/cm^2^ Sham ESWT same timing as for rESWT group	Pain (pressure pain threshold PPT, pain score, VAS) QoL (SF-36) Hospital anxiety and depression scale (HADS)	T0: Before treatment T1: 2 weeks later the end of treatment T2: 4 weeks after the end of treatment	rESWT is as effective as US. rESWT and US are significantly more effective than sham rESWT in reducing pain and improving QoL but make no difference in HADS.
Rahbar et al. 2021 [16]	RCT	Neck, Upper back	72 (24 rESWT + exercise, 24 US + hot pack + self-stretch-exercises, 24 self-stretch-exercises)	SW: 2000 pulses EFD: 60 mJ/m^2^ 5 Hz T: once a week for 4 weeks	Group 2 US + hot pack + self-stretch-exercises Group 3 self-stretch-exercises	Pain (PPT, VAS) Disability (NDI)	T0: before treatment T1: first week of treatment T2: fourth week of treatment	rESWT and US were equally effective in improving pain and reducing disability and were significantly more effective than control.
Gezginaslan et al. 2020 [17]	RCT	Neck and shoulder	94 (49 rESWT, 45 superficial hot pack + TENS + US)	SW: 1500 to 4500 pulses EFD: 0.26 mJ/mm^2^. T: Seven sessions with three days interval	Superficial hot pack, TENS, and US were administered five times a week for two weeks. Continuous US at 1 Mhz was applied at a dose of 1.5 watt/cm^2^ for six minutes daily. TENS was applied for 30 min, and HP was applied 20 min daily.	Pain (VAS) Sleep (Pittsburgh Sleep Quality Index—PSQI) Fatigue (Fatigue Scale) Disability (Functional Assessment of Chronic Illness Therapy—FACIT, NDI) Depression (Beck Depression Inventory—BDI) QoL (SF-36)	T0: before interventions T1: after one month of interventions	rESWT was more effective than a combination of hot packs, TENS, and US in reducing pain and improving sleep quality, disability, depression, and QoL.
Taheri et al. 2021 [18]	RCT	Upper trapezius	37 (18 rESWT, 19 Phonophoresis)	SW: 2000 pulses EFD: 0.2 mj/mm^2^ with 10 Hz frequency T: three sessions once a week for three weeks	Phonophoresis with hydrocortisone gel 1%, 1 MHz frequency, and 1.2 Wt/cm^2^ power over the trigger points on the trapezius muscle for 10 min. Three times a week for three weeks	Pain (VAS) Disability (NDI)	T0: before the first Session T1: and one week after the second session	Both phonophoresis and rESWT effectively decreased pain and neck disability with the superiority of rESWT.
Walsh et al. 2019 [19]	pilot RCT	Thigh (quadriceps)	21 (7 rESWT; 7 DN; 7 control group)	SW: 1000 pulses at 20 Hz EFD: up to 5 bars T: 3 sessions per week Surrounding tissue was treated with 2000 pulses at 20 Hz up to 3 bars	DN: acupuncture needle in the most painful TrPs in Vastus Lateralis and Vastus Medialis or control from 30 s to 2 min Control: rest for 7 min in each of the four positions used to measure PPT.	pain (PPT measured with algometer)	T0: baseline T1: at 23–25 days T2: 28 days	rESWT and DN were both effective in reducing pain, but DN can be associated with post-treatment soreness.
Luan et al. 2019 [20]	RCT	Neck (upper trapezius)	65 (32 rESWT; 33 DN)	SW: 2000 pulses EFD: 0.10 mJ/mm^2^ T: once a week for 3 weeks	DN into MTrPs for 10 s once a week for three weeks	Pain (VAS, PPT) Disability (NDI), and shear wave ultrasound elastography of the upper trapezius MTrPs	T0: baseline T1: 15–30 min after the first treatment T2: 1 month after treatment T3: at 3 months after treatment	rESWT and DN were both effective in reducing pain and disability and in reducing the shear modulus of myofascial trigger points.
Manafnezhad et al. 2019 [21]	RCT	Neck (upper trapezius)	70 (35 rESWT; 35 DN)	SW: 1000 pulses EFD: 60 mj, 16 Hz T: once a week for 3 weeks	DN with fast-in and fast-out needling technique (1–2 min)	Pain (PPT, NPRS) Disability (NDI)	PPT and NPRS (0–10) were assessed before each treatment session and one week after last session; NDI before first treatment and one week after last session	rESWT and DN were equally effective in reducing pain and disability.
Eftekharsadat et al. 2020 [22]	RCT	Low Back (quadratus lumborum)	54 (27 rESWT; 27 corticosteroid trigger point injection—TPI)	SW: 1500 pulses/session EFD: 0.1 mJ/mm^2^ /min, frequency of 10–16 Hz, and pulse rate of 160/min in total	TPI of 40 mg triamcinolone + 2 mL of lidocaine 2%	Pain (VAS, PPT) Disability (ODI) QoL (SF36)	T0: before interventions T1: after two weeks from treatment T2: after four weeks of treatment	Corticosteroid TPI was more effective than rESWT in reducing pain and disability in the short term. However, rESWT was more effective in reducing pain and disability and improving QoL at 1 month.
Li and Wu 2020 [23]	Case-control study	TMJ	80 (40 rESWT; 40 ultrashort wave—UW)	SW: 1000–1500 pulses EFD: 8 Hz frequency T: once a week for four weeks	UW was applied by placing the electrodes 2 to 3 cm to the mandibular joint, and each treatment lasted 15 min once a day for 5 a week for 4 weeks.	Pain (VAS); Pain-free maximum mouth opening (MMO); Friction index: mandibular movement (MM), joint noise (JN), joint press (JP), and disability index (DI).)	T0: before the treatment T1: four weeks after therapy	rESWT was more effective than UW in reducing pain and improving functional indexes of temporomandibular joint and mouth.
Sugawara et al. 2021 [24]	Retrospective study	MPS or AP	1580	According to clinician experience (1983 ± 406.5 pulses/session, 14.00 ± 2.05 Hz and 2.5 ± 0.5 bar) for two sessions	None	Pain (VAS)	T0: before the first Session T1: and one week after the second session	rESWT decreased pain above all in patients with intense myofascial pain (VAS > 70 mm).

Abbreviations: ESWT: Extracorporeal Shock Wave Therapy; SW: shock-waves number; EFD: energy flux density; T: treatment sessions; TPI: trigger point injection; PPT: pain pressure threshold; MPQ: McGill Pain Questionnaire: PRS: Pain Rating Scale; VAS: Visual Analogic Scale; ODI: Oswestry Disability Index; NDI: Neck Disability Index: QoL: Quality of Life: DN: Dry Needling; SPADI: Shoulder Pain and Disability Index; US: Ultrasound; MTrP: Myofascial trigger point: FACIT: Functional Assessment of Chronic Illness Therapy; BDI: Beck Depression Inventory; PSQI: Pittsburgh Sleep Quality Index: UW: Ultrawaves; TMJ: Temporomandibular joint; MPS: Myofascial Pain Syndrome; AP: Articular Pain.

**Table 4:** Main characteristics and key findings of the included studies about Focused ESWT for myofascial pain syndrome.

Author, Year	Study Design	Site of Application	Sample Size: Total (Group)	Intervention (ESWT Protocol)	Control	Outcomes	Timing	Main Findings
Jeon et al. 2012 [25]	RCT	Neck (trapezius)	30 (15 × 2 groups)	SW: 1500 pulses EFD: 0.10 mJ/mm^2^ T: once a week for 3 weeks	TPI treatments and 5 TENS treatments were given 5 times a week with a duration of 20 min a day.	Pain (VAS, PRS, MPQ) Neck ROM	T0: before first therapy T1: after first therapy T2: after third therapy	No significant between-group differences were found for pain (VAS, MPQ, and PRS) and ROM at 1 week after the first and third treatment.
Ji et al. 2012 [26]	RCT	Neck (upper trapezius)	20 (9 fESWT groups vs. 11 in the control group)	SW: 1000 pulses EFD: 0.056 mJ/mm^2^ T: twice a week for 4 sessions	Ineffective ESWT (0.001 mJ/mm^2^).	pain (VAS, PPT);	T0: baseline T1: right after fourth treatment	Intervention significantly reduced pain (VAS) and increased PPT compared to control group.
Park et al. 2018 [27]	RCT	Neck (upper trapezius)	30 (15 × 2 groups)	SW: 1500 pulses EFD: 0.210 mJ/mm^2^ T: once a week for 2 weeks	SW: 1500 pulses EFD: 0.068 mJ/mm^2^ T: once a week for 2 weeks	Pain (VNS, pain threshold) Disability (NDI) Neck ROM	T0: before treatment T1: after treatment	High-energy ESWT was more effective than low-energy ESWT in improving NDI score and neck flexion ROM at 2-week follow-up
Kamel et al. 2020 [28]	RCT	Neck (upper trapezius)	46 (23 × 2 groups)	SW: 1000 pulses EFD: 0.25 mL/mm^2^ T: once a week for 4 weeks + Topical 1% diclofenac gel (3 times/day for 4 weeks)	Only topical 1% diclofenac gel (3 times/day for 4 weeks)	Pain (VAS, PPT) Neck ROM	T0: baseline T1: after 2 weeks from treatment T2: after 4 weeks from treatment	Intervention showed a significant improvement in pain (VAS and PPT) and ROM (lateral bending and rotation bilaterally) compared to control group in patients with MPS after neck dissection surgery at 4 weeks.
Moghtaderi et al. 2014 [29]	RCT	Gastrocnemius–soleus; heel region	40 (20 × 2 groups)	SW: 3000 + 400 each trigger point EFD: 0.2 mJ/mm^2^ T: three sessions every week	SW: 3000 pulses EFD: 0.2 mJ/mm^2^ on heel region T: three sessions every week	Pain (VAS) Disability (Roles and Maudsley score, RM)	T0: baseline T1: eight weeks after treatment	Intervention was more effective than control for improvement of pain and activity (VAS and modified RM score) at 8 weeks follow-up
Hong et al. 2017 [30]	Retrospective study	Quadratus lumborum	30 (15 × 2 groups)	SW: 2000 pulses EFD: 0.085–0.148 mJ/mm^2^ T: three times at 3-day interval	TPI three times at the tender point at 3-day intervals	Pain (VAS, PPT) Disability (ODI, Roles and Maudsley RM, Quebec Back Pain Disability Scale QBS)	T0: before the initial treatment T1:immediately after the third treatment T2: 1 month after treatment	Intervention was more effective than control for pain reduction (VAS and PPT) immediately after treatment and at 1-month follow-up; no statistically significant between-group differences were found for disability indexes (ODI, RM score, QBS).
Ümit Yalçın 2021 [31]	Retrospective study	Neck (upper trapezius)	262 (75 ESWT exercise group, 82 KT + exercise group, 105 exercise group)	SW:1500 pulses EFD: 0.056 mJ/mm^2^ T: three sessions every week	X-shaped KT (2 bands of 7.5 cm long I tape glued one after the other, crossing each other) applied every four days for a total three times in twelve days by the same physician	Pain (PPT, VAS) Disability (NDI) Neck ROM	T0: baseline T1: after three months from treatment	Intervention was significantly more effective than KT and control in reducing pain and increasing PPT, NDI score and controlateral lateral flexion

Abbreviations: ESWT: Extracorporeal Shock Wave Therapy; SW: shock-waves number; EFD: energy flux density; T: treatment sessions; TPI: trigger point injection; PPT: pain pressure threshold; MPQ: McGill Pain Questionnaire: PRS: Pain Rating Scale; VAS: Visual Analogic Scale; RM: Roles and Maudsley; CS: Corticosteroids; ODI: Oswestry Disability Index; QBS: Quebec Back Scale; NDI: Neck Disability Index; KT: Kinesiological Taping.

## Data Availability

Not applicable.
